# The Destruction of Shenanegan

**Published:** 1885-09

**Authors:** 


### THE DESTRUCTION OF SHENANEGAN.

(*After Byron: "The Destruction of Sennacherib."*)

The Chairman came forward so gallant and bold,
And his shirt-front was gleaming with diamonds and gold;
The smile on the faces was fearful to see,
As he read the report of that deep commit-tee.
The appointments they made were like the sands on the shore;—
They gave all to their friends—and would gladly give more,—
Ignoring the members from the west and the south,
Which brought forth destruction from Shoemaker's mouth.
He told of the bargains with new-coders made—
The plans and manœuvres that Jacobi laid
To capture the Congress in this mighty nation,
By an extra-sharp game on the *As*-so-ci-ation!
Then the fire from their eyes, like a sword from its sheath,
Flashed forth in its vengeance; and gnashing their teeth,
They glared like a demon on the men from the South,
And the impotent froth-foam rose white on the mouth.
But the Angel of Justice spread his wings on the blast,
And whispered "no go," to those men as he passed.
Then the thought of the schemers was like the poison in the cup;
Because thus defeated they'd "bust the thing up!"
And the Doctors of Delphia are loud in their wail,
Because right, and justice, and *ethics* prevail.
Thus the game of Shenanegan the profession soon saw
Would never "hold water," in the eyes of the law.

